# Recognition of Predator Type and Risk Level in Azure‐Winged Magpies (*Cyanopica cyanus*) Through Visual and Auditory Cues

**DOI:** 10.1002/ece3.70749

**Published:** 2024-12-16

**Authors:** Taijun Zuo, Jiaojiao Wang, Jiangnan Liu, Jianping Liu, Qindong Zhou, Jianhua Hou

**Affiliations:** ^1^ College of Life Science Hebei University Baoding China; ^2^ Engineering Research Center of Ecological Safety and Conservation in Beijing–Tianjin–Hebei (Xiong'an New Area) of MOE Baoding China; ^3^ Hebei Basic Science Center for Biotic Interaction Baoding China; ^4^ College of Biological Sciences and Engineering North Minzu University Yinchuan China; ^5^ School of Life Sciences Guizhou Normal University Guiyang China

**Keywords:** anti‐predation, auditory recognition, azure‐winged magpie, predation risk, visual recognition

## Abstract

Predation events are an important key factor determining the survival and reproduction of prey species. To cope, prey species have evolved various anti‐predator strategies, including mechanisms for accurate predator identification and distinguishing predator types and risk levels. Birds rely on visual, auditory, and olfactory cues to perceive and categorize predators. However, previous studies have focused on single sensory modalities and have largely been conducted during breeding seasons. Thus, analyses of the accuracy and differences in predator recognition cues, especially during non‐breeding periods, are needed. In this study, predator recognition in Azure‐winged Magpies ( *Cyanopica cyanus* ) was evaluated during the non‐breeding season. We examined responses to two predators, Common Kestrel ( *Falco tinnunculus* ) and Domestic Cat ( *Felis catus* ), and two non‐predators, Oriental Magpie (*Pica serica*) and Oriental Turtle Dove ( *Streptopelia orientalis* ). Using specimens and playback experiment, the ability of Azure‐winged magpies to identify threats through both visual and auditory cues was evaluated. The results showed that Azure‐winged Magpies can identify predator types through both visual and auditory cues, accurately distinguish threat levels, and adjust their foraging behavior accordingly. Notably, they exhibited the strongest anti‐predator response to Domestic Cats (frequently encountered under natural conditions), as evidenced by longer observation times, shorter foraging duration, and quicker flight responses. The results indicate that Azure‐winged Magpies can correctly identify predator types and threat levels through both visual and auditory cues and exhibit stronger anti‐predator behaviors with increasing apparent threat levels.

## Graphical abstract

In this study, we demonstrated that Azure‐winged Magpies ( *Cyanopica cyanus* ) uses both visual and auditory cues to detect different predator types and to assess the risk level during the non‐breeding season. In particular, a stronger anti‐predator response was observed to Domestic Cats than to the less frequently encountered Common Kestrel and two non‐predator species, Oriental Magpie and Oriental Turtle Dove. These findings demonstrate the complex mechanisms by which magpies adjust foraging behavior in response to specific predators.

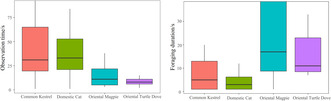

## 1 Introduction

Predation is a major cause of death for most animals in the wild (Sinclair et al. 2003); it is one of the primary selection pressures driving the evolution of prey species (Caro 2005). Prey species have evolved a variety of anti‐predator strategies, including warning coloration, visual polymorphism, tail signaling, alarm calls, predator distraction behaviors, and mobbing (Shang 2014). However, excessive time spent on anti‐predator behaviors can consume energy and reduce opportunities for foraging and reproduction (Lima 1998). According to the threat‐sensitive predator avoidance hypothesis, the abilities to balance predation risk and foraging opportunities and to adjust anti‐predator behaviors are crucial for animal survival and for maximizing fitness (Lima and Dill 1990). Accurate identification of predators and non‐predators as well as distinguishing between predator types and risk levels are critical first steps in anti‐predator strategies (Sam and Fuchs 2011).

Birds species typically use visual, auditory, and olfactory cues to perceive and identify predator types (Billings, Greene, and De La Lucia Jensen 2015). Research on visual recognition has focused mainly on the recognition of predator size, morphology, type, and behavior. The allometric risk hypothesis suggests that birds of prey of similar or slightly larger body size compared with potential prey pose a greater predation risk than that of birds that are significantly larger or smaller (Templeton, Greene, and Davis 2005). For instance, when faced with three predators of different sizes (the Mountain Pygmy Owl *Glaucidium gnoma* , Sharp‐shinned Hawk *Accipiter striatus* , and Eurasian Goshawk *Accipiter gentilis* ), Steller's Jay ( *Cyanocitta stelleri* ) exhibited significant differences in interruption times during feeding. The longest interruption occurred in response to the Sharp‐shinned Hawk, which was closest in size to the jay (Billings, Greene, and MacArthur‐Waltz 2017). In addition to predator size recognition, morphological recognition is also an important means of identification, and many birds have gaze sensitivity, that is, the ability to respond to the presence, orientation, or movement of the head and eyes (Carter et al. 2008; Davidson and Clayton 2016). Furthermore, they can recognize not only the direction of the head but also the subtle differences in eye direction, which have been reported in birds, mammals, reptiles, and fish, and gaze may be associated with predator aggression (Davidson and Clayton 2016). Predator behavior also profoundly affects the anti‐predator behavior of prey. Based on experimental manipulation of robotic predators, Blue Tits ( *Cyanistes caeruleus* ) not only respond to the presence of sparrowhawks, but also change specific anti‐predator behaviors when predators perform different behaviors, which indicates that prey will pay attention to predator’ status and behavior (Carlson, Healy, and Templeton 2017).

Regarding predator type, depending on the targeted prey (eggs, nestlings, or adult), the defensive behaviors exhibited by prey also vary. The Great Reed Warbler ( *Acrocephalus arundinaceus* ), which can identify the sparrowhawk as a predator, shows a significantly stronger response to attacks from the parasitic cuckoo than to those from sparrowhawks. When a sparrowhawk is near the nest, the Great Reed Warbler exhibits less aggressive behavior and behaves more cautiously to reduce the risk of being preyed upon (Trnka and Prokop 2012). Depending on the habits of predators, they can be classified into ground predators or aerial predators. The threat level and response intensity vary. For example, the Mustached Warbler ( *Acrocephalus melanopogon* ), which nests at low heights, exhibits a stronger response (including the most frequent alarm calls and the longest delay in returning to the nest) to snakes than to the Beringian Ermine ( *Mustela erminea* ) and Western Marsh Harrier ( *Circus aeruginosus* ). In contrast, the Great Reed Warbler, which nests at greater heights, shows the most frequent alarm calls and longest delay in returning to the nest in response to the Western Marsh Harrier (Kleindorfer, Fessl, and Hoi 2005). Some bird species even exhibit different response intensities to different types of aerial predators. For example, the House Wren ( *Troglodytes aedon* ) can distinguish predation risks posed by three aerial predators, with significantly lower nest entry frequency and longer return times in response to the Roadside Hawk ( *Buteo magnirostris* ) than to the Chimango Caracara ( *Milvago chimango* ) and Double‐toothed Kite ( *Harpagus bidentatus* ) (Duré Ruiz, Mariana, and Fernández 2017).

Furthermore, in complex external environments, auditory cues are crucial for some birds to identify predators, especially for cavity‐nesting species where visual recognition is limited (Schneider and Griesser 2012). Auditory recognition can directly occur through predator calls. For example, the female Yellow‐rumped flycatcher is able to distinguish between the calls of the Oriental Turtle Dove and Eurasian Sparrowhawk and responds to the call of the sparrowhawk by fleeing the nest (Yue 2019). Birds can also respond to conspecific alarm calls or even eavesdrop on the alarms of other species (Billings, Greene, and De La Lucia Jensen 2015; Billings, Greene, and MacArthur‐Waltz 2017). For instance, the White‐browed Scrubwren ( *Sericornis frontalis* ) and Superb Fairywren ( *Malurus cyaneus* ) respond to each other's alarm calls by fleeing (Fallow and Magrath 2010).

Finally, recognition by smell is also an important strategy for predator identification, especially for ground‐dwelling species, particularly because the main predators of birds, such as mammals and reptiles, use chemical signals for communication. For example, the Red‐legged Partridge ( *Alectoris rufa* ) shows reduced selection and sand‐bathing behavior in response to sand‐bathing trays treated with Domestic Ferret ( *Mustela putorius furo* ) feces and Eurasian Hoopoe ( *Upupa epops* ) alarm secretions, with no significant differences between the two cues. In contrast, a sand bath containing orange oil had no effect on partridge behavior (Mahr and Hoi 2018). Additionally, some studies have used multiple stimuli to test prey’ anti‐predator behavior. For example, the Steller's jay ( *Cyanocitta stelleri* ) and the British tit combine visual and auditory cues to assess predation risk, encoding this risk information in their alarm calls (Billings, Greene, and MacArthur‐Waltz 2017; Carlson, Healy, and Templeton 2017). The three‐spined stickleback ( *Gasterosteus aculeatus* ) can evaluate predation risk based on visual or olfactory cues and adjusts its behavioral responses accordingly, showing a stronger reaction to visual cues (Landeira‐Dabarca et al. 2019). However, more research has focused on single signals, such as visual cues, which are often concentrated during the breeding season when birds are typically solitary or in pairs. In contrast, many bird species gather in flocks during the non‐breeding season, and whether their perception and response to risk differs with increasing group size still requires further research.

Azure‐winged Magpies, which belongs to the order Passeriformes and the family Corvidae, are distributed primarily in East Asia (Valencia, Cruz, and González 2003). During the non‐breeding season, magpies exhibit strong social and social behavior (Valencia, Cruz, and González 2003), which makes it easy to group them and thus conduct experiments on them in each location. In addition, it is the dominant species in the study area in the present study (campus), and various predators may pose different risks to magpies in the location. Therefore, Azure‐winged Magpie is an ideal species for studying the perception of predation risk and anti‐predator behavior in non‐breeding season birds. When facing predators, magpies may have to strike a balance between offspring safety and their own safety in the breeding season, while in the non‐breeding season, magpies only need to consider their own safety to perform anti‐predation behavior. Moreover, in the breeding season, the anti‐predation behavior of magpies is influenced by breeding stage (Wang et al. 2021). In addition, during the breeding season, magpie’ nest position in the present study site is higher; therefore, the experimental operation are more difficult than those in the non‐breeding season.

The aim of the present study was to investigate how Azure‐winged Magpies ( *Cyanopica cyanus* ) recognize and respond to various predators during the non‐breeding season. Using both specimen and audio playback experiments, the ability of Azure‐winged Magpies to differentiate predators through visual and auditory cues was examined. The specimens selected for the experiment included the simulated model of Domestic Cat (the most common ground predator on campus), which is a stuffie cat bought from an online store with almost the same size and appearance as a real cat; and taxidermy models of three bird species, including the Common Kestrel (*Falco tinnunculus*) (the aerial predator), Oriental Magpie (*Pica serica*) (a food or territorial competitor of the Azure‐winged Magpie), and Oriental Turtle Dove ( *Streptopelia orientalis* ) (a neutral control). A playback experiment was performed using the sounds of the four species. By comparing results obtained using specimens and audio playbacks, the present study assessed the accuracy and differences between visual and auditory recognition in the birds. In particular, the following hypotheses were evaluated: (1) the Azure‐winged Magpie can perceive predation risk through both visual and auditory cues, (2) the Azure‐winged Magpie can accurately distinguish threat levels using both visual and auditory recognition and adjust its behavior accordingly, and (3) due to the higher rate of encounters and greater threat level posed by Domestic Cats on campus, the Azure‐winged Magpie will exhibit a stronger response to Domestic Cats than to Common Kestrels.

## 2 Materials and Methods

### 2.1 Study Site and Subjects

The field study was conducted from December 2023 to February 2024 on the campus of Hebei University (38°86′ N–38°88′ N, 115°51′ E–115°56′ E). Common plants on campus include Lily tree ( *Magnolia denudata* ), Chinese Wild Peach (*Prunus davidiana*), Black Locust ( *Robinia pseudoacacia* ), Pomegranate ( *Punica granatum* ), Ginkgo ( *Ginkgo biloba* ), and Deodar Cedar ( *Cedrus deodara* ). Common birds include the Oriental Magpie, Eurasian Tree Sparrow ( *Passer montanus* ), Azure‐winged Magpie, and Oriental Turtle Dove, while the Common Kestrel being less numerous. We observed more than 20 colonies of Azure‐winged Magpie, ranging in size from 5 to 40 individuals, on campus, each of which was followed throughout the day and confirmed to have separate territories.

### 2.2 Attracting Azure‐Winged Magpies

Before the experiment began, a total of 20 numbered feeding stations (26 cm × 36 cm; in subsequent data analysis, some experiments at feeding stations were omitted due to interference) were set up in areas with abundant Azure‐winged Magpies on campus, such as woodlands and gardens. Each station was fixed on top of a 1.5 m tall stand and spaced at least 200 m apart. In the course of observation before the experiment, it was found that they have their discrete territories. Bird food (Jiangsu Synergetic Pharmaceutical Bioengineering Co. Ltd., Nanjing, China) was replenished twice daily on feeding trays on stands (1.5 m high) in the morning and evening. Cameras were placed near the feeding stations to record the feeding behavior of Azure‐winged Magpies throughout the day, and the peak times of daily feeding were recorded to facilitate subsequent experiments. Feeding was conducted consistently for at least a half a month to establish stable feeding stations.

### 2.3 Preparation of Sound Playbacks

**FIGURE 1:**
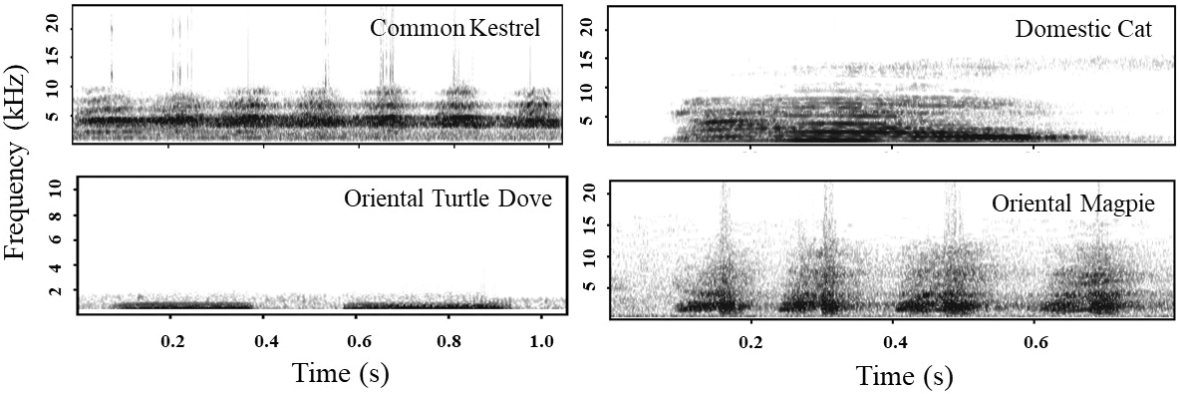
Spectrograms of the sound types used in the playback experiment.

In November 2023, vocalizations of Domestic Cats were recorded on campus, with a total of 10 audio clips obtained. The vocalization audio clips for the Common Kestrel, Oriental Magpie, and Oriental Turtle Dove were downloaded from Xeno‐Canto recordists (http://www.xeno‐canto.org/). Call sounds with good recording quality were selected, and three sounds were selected for each species to minimize pseudoreplication. One minute of background noise was extracted from the downloaded sounds as a control (Playback of the background noise did not reveal any response from the birds; therefore, it was not included in further analysis). A total of four types of sounds (Domestic Cats, Common Kestrel, Oriental Magpie, and Oriental Turtle Dove) were obtained (Figure 1). Using Raven Pro 1.4 (https://www.ravensoundsoftware.com/software/raven‐pro/), low‐frequency noise was removed, and parts of the recordings that overlapped with other bird species were deleted. Each sound was edited to 1 min for playback and saved in WAV format. Playback was conducted using Bluetooth speakers, with the playback volume standardized to approximately 65 dB at a distance of 1 m.

### 2.4 Specimen Experiments

The specimen experiments were conducted from December 2023 to February 2024, during the non‐breeding season of the Azure‐winged Magpie. Four specimen were used: Common Kestrel (aerial predator), Domestic Cat (ground predator), Oriental Magpie (territorial competitor), and Oriental Turtle Dove (control). All specimens were placed in a natural standing posture. To avoid errors due to pseudoreplication, two specimens or models of each type were selected, and one was randomly chosen for each experiment. The effects of different specimens were tested and no significant differences in responses were found, indicating that the choice of specimens did not influence the results. The experiments were conducted on clear, windless days from 8:00 to 17:00. One type of specimen was randomly selected and fixed on a stand approximately 3 m from the feeding station (stand height, 1.5 m). A voice recorder (Sony PCM‐A10, Japan) was used to record any alarm calls made by the Azure‐winged Magpies. A digital video (Sony FDR‐AX45) was fixed approximately 10 m from the feeding trays, with the focal length adjusted to clearly record the feeding station, specimen, and surrounding environment for later data verification and correction. Following setup, the researchers promptly concealed themselves at approximately 15 m from the feeding trays for observations. When an Azure‐winged Magpie was observed within 5 m of the feeding station, the timing started. During this period, the behavior of the Azure‐winged Magpies was observed, and the following information was recorded: (1) number of Azure‐winged Magpies in the group; (2) time from when the Azure‐winged Magpie was detected within 5 m of the specimen until it started foraging (observation time); (3) duration of foraging; (4) alarm behavior of the Azure‐winged Magpies.

After each trial, a different model or specimen type was randomly selected for the next experiment. Four specimens were placed in each position. However, due to interference and other factors, some experiments were terminated, and the specific samples obtained are shown in the results. A minimum interval of 3 h was maintained between trials using different specimen types. Each feeding station was tested with each type of model or specimen only once.

### 2.5 Playback Experiment

From January to February 2024, playback experiments were conducted using the four types of sounds mentioned above. The experiments were carried out on clear, windless days. The speaker controlled by Bluetooth was fixed on a stand approximately 3 m from the feeding tray (stand height, 1.5 m). The digital video was fixed approximately 10 m from the feeding tray, with the focal length adjusted to clearly record the feeding tray, speaker, and surrounding environment. The experimenter moved quickly to a concealed location about 15 m from the tray. Once an Azure‐winged Magpie arrived at the feeding point and began to forage, the playback experiment started. At each feeding station, one of the four types of sounds was selected randomly for playback. The order of sound playback was randomized. The following information was recorded: (1) the number of Azure‐winged Magpies in the group; (2) behavior of the Azure‐winged Magpies (response intensity), such as no response, alarm, or fleeing; (3) reaction time (i.e., the time until an alarm call or fleeing occurred; if there was no reaction, the reaction time was recorded as 60 s).

After each trial, a different sound was selected for the next experiment. To mitigate potential influence, a minimum interval of 3 h was maintained between trials. Each feeding station was tested with each type sound only once.

### 2.6 Statistical Analyses

A generalized linear mixed model (GLMM, glmmTMB in R(4.1.3)) was used to analyze whether Azure‐winged Magpies recognize different predators. The three response variables recorded were used as target variables: observation time within 5 m, foraging time (Poisson distribution), and whether alarm calls were made (binomial distribution). Specimen type and group size were used as fixed effects, and station identity was used as a random effect. We proceeded to perform pairwise post hoc comparisons between levels of statistically significant control predictors, utilizing the ‘emmeans’ and ‘multcomp’ package.

Since the response intensity to different intruder sounds in the playback experiment was an ordinal categorical variable, cumulative link mixed models (CLMMs, clmm in the R package ordinal) were used to analyze differences in the response intensity of Azure‐winged Magpies to different intruder sounds. In this model, response intensity scores were used as the target variable, different types of playback sounds and group size as fixed effects, and the station identity as a random effect. Additionally, a GLMM (glmmTMB in R(4.1.3)) was also constructed to analyze whether the response time of Azure‐winged Magpies differed with respect to different predator sounds. In this model, response time was used as the target variable (Poisson distribution), with specimen type and group size as fixed effects and the station identity as a random effect. A two‐tailed likelihood ratio test was then used for comparisons. When significant differences in treatments were observed, separate CLMMs for each treatment were carried out. To mitigate the order effect, the specimen presentation sequence was designated as the independent variable in the specimen experiment, while the observation and feeding durations were treated as continuous response variables. The Kruskal–Wallis test was employed to assess differences among groups. In the playback experiment, the playback sequence served as the independent variable, with reaction time as the continuous response variable; again, the Kruskal–Wallis test was used to evaluate inter‐group differences. Furthermore, response intensity was analyzed using the Chi‐squared test to examine differences among groups. All tests were two‐tailed, and the significance level was *p* < 0.05.

## 3 Results

### 3.1 Specimen Experiments

When conducting specimen experiments at 20 feeding stations, the experiments were abandoned due to sudden human interference or passing vehicles during the experiment. Data on the placement of cat, kestrel, and magpie specimens were obtained successfully at 14 feeding stations, seven of which were dove specimens. That is, specimens of three types of cat, kestrel, and magpie were displayed for each of the 14 flocks of magpies, seven of which also displayed dove specimens.

The GLMM revealed that both specimen type (*χ* ^2^ = 265.82, df = 3, *p* < 0.001) and group size (*χ* ^2^ = 284.34, df = 1, *p* < 0.001) had a significant impact on observation time within 5 m and foraging duration. However, neither specimen type (*χ* ^2^ = 2.69, df = 3, *p* = 0.44) nor group size (*χ* ^2^ = 3.31, df = 1, *p* = 0.07) had a significant impact on whether alarm calls were made (Table 1).

**TABLE 1:** Generalized linear mixed model of Azure‐winged magpie responses to different specimens.

Variable	$x^{2}$	*df*	*p*
Observation time within 5 m			
Specimen type	265.82	3	< 0.001
Group size	284.34	1	< 0.001
Foraging duration			
Specimen type	228.70	3	< 0.001
Group size	297.37	1	< 0.001
Alarm behavior			
Specimen type	2.69	3	0.44
Group size	3.31	1	0.07

**FIGURE 2:**
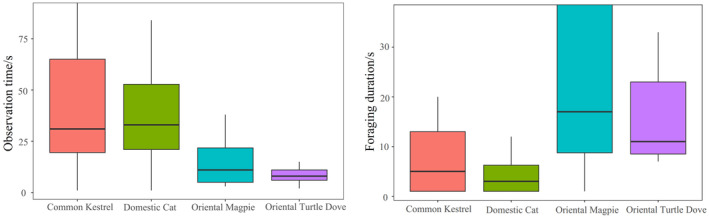
Responses of Azure‐winged magpies to different specimens.

**FIGURE 3:**
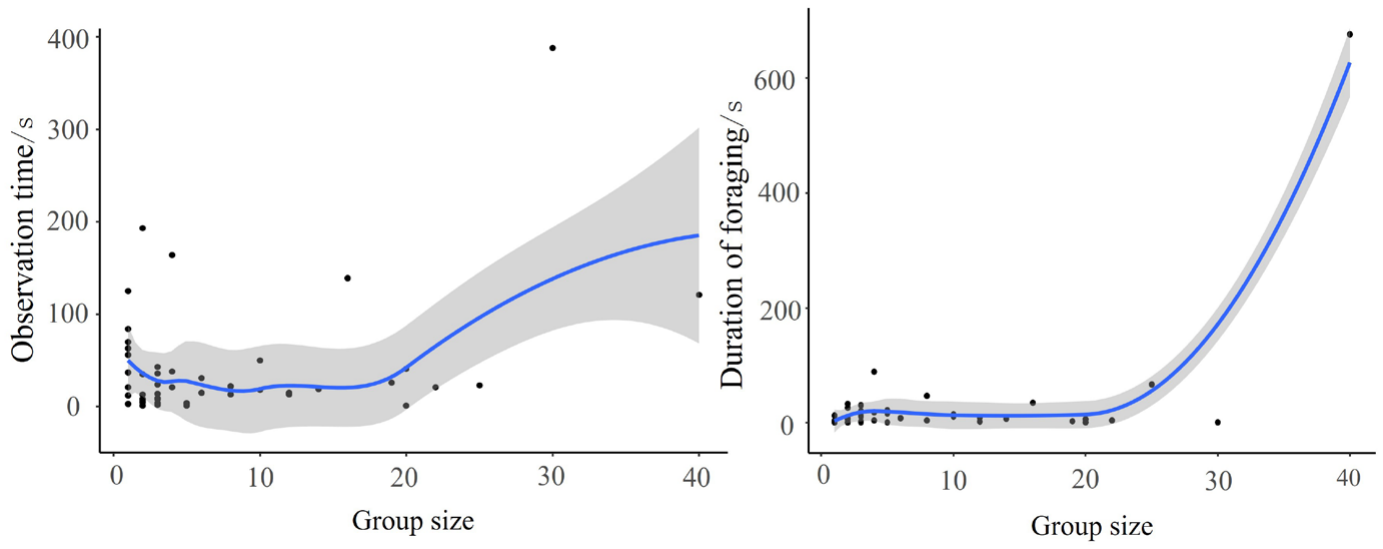
Relationship between group size and response of Azure‐winged magpies when faced with specimens.

In pairwise comparisons, observation time of Azure‐winged Magpies in response to the Common Kestrel (emmean ± SE: 3.50 ± 0.21; the same below) specimen was significantly shorter than that in response to the Domestic Cat specimen (3.78 ± 0.21) (*z* = −4.69, *p* < 0.001). However, observation time in response to the Common Kestrel specimen was significantly longer than those in response to the Oriental Magpie (3.07 ± 0.21) (*z* = 7.18, *p* < 0.001) and Oriental Turtle Dove (1.73 ± 0.25) (*z* = 12.82, *p* < 0.001). Observation time in response to the Domestic Cat specimen was also significantly longer than those for the Oriental Magpie (*z* = 9.64, *p* < 0.001) and Oriental Turtle Dove (*z* = 14.70, *p* < 0.001). Additionally, observation time in response to the Oriental Magpie specimen was significantly longer than that for the Oriental Turtle Dove (*z* = 9.64, *p* < 0.001). When the target variable was foraging duration, there was no significant difference in foraging time of Azure‐winged Magpies between the Common Kestrel and Domestic Cat specimens (*z* = 0.03, *p* = 0.97). However, foraging duration after seeing the Common Kestrel (1.68 ± 0.30) and Domestic Cat specimens (1.67 ± 0.30) were significantly shorter than those after seeing the Oriental Magpie (3.05 ± 0.29) and Oriental Turtle Dove specimens (2.32 ± 0.30) (*p* < 0.001). Furthermore, foraging duration in response to the Oriental Magpie specimen was significantly longer than that for the Oriental Turtle Dove (*z* = 6.14, *p* < 0.001) (Figure 2). Additionally, group size of Azure‐winged Magpies affected both the observation time within 5 m and the foraging duration. The larger the group size, the longer the observation time within 5 m and the foraging duration (Figure 3). We did not find any effect of the order effect on the response variable (All *p* > 0.05).

### 3.2 Playback Experiment

As with the specimen experiment, it was difficult to play all four sounds to the magpies at each of the 20 feeding stations. In this playback experiment, we only played all four types of sounds to the four feeding stations (namely 4 flocks of magpies). In 14 flocks, the magpies received playback of sounds from kestrel, cat and magpie. In addition, seven flocks of magpies received playback of sounds from the turtle dove.

**FIGURE 4:**
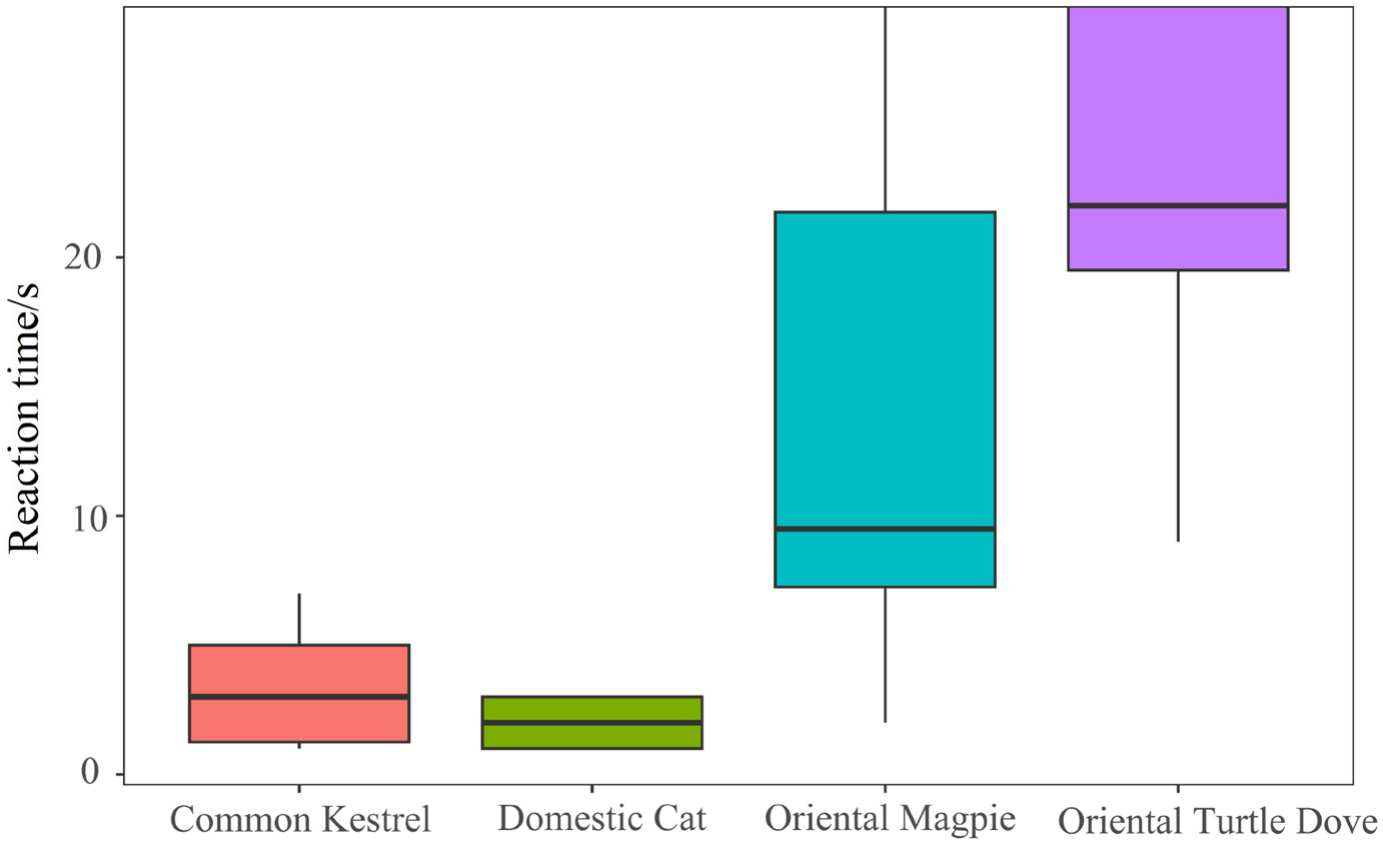
Response times of Azure‐winged magpies to different intruder Sounds.

**FIGURE 5:**
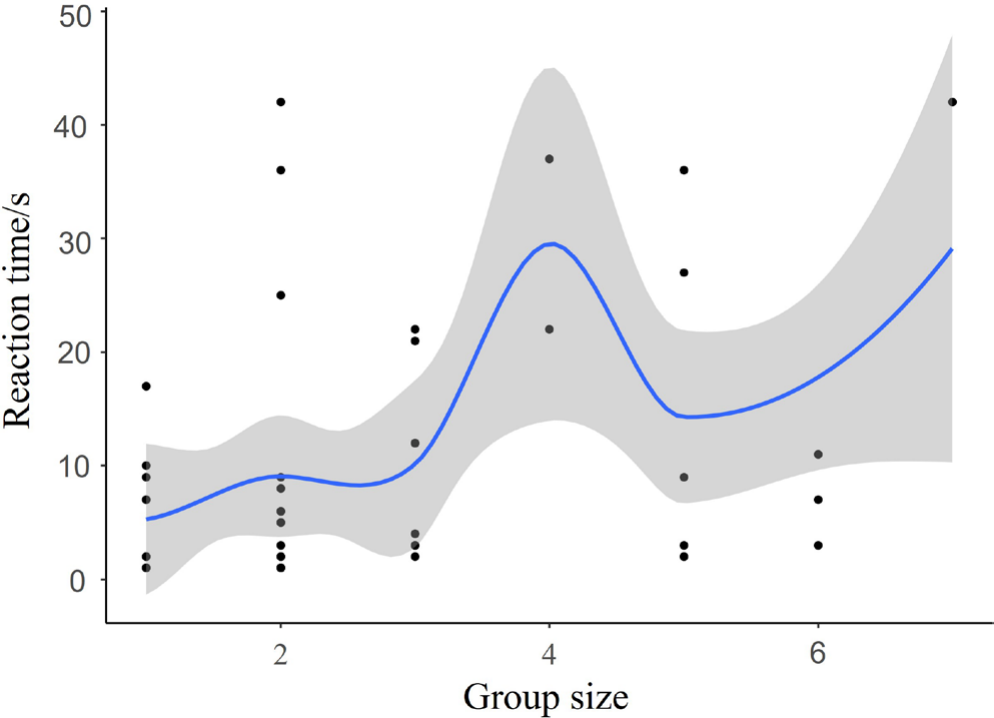
Relationship between group size and response times of Azure‐winged magpies.

The GLMM results indicated that both the type of playback sound ($x^{2}$ = 193.56, *p* < 0.001) and group size ($x^{2}$ = 32.30, *p* < 0.001) significantly affected the reaction time of Azure‐winged Magpies (Table 2). In post hoc pairwise comparisons, there were no significant differences in reaction time between the playback of Common Kestrel (1.27 ± 0.19) and Cat (1.11 ± 0.19) sounds (*z* = 0.73, *p* = 0.47). However, the reaction time for both Common Kestrel and Cat sounds was significantly shorter than for Oriental Magpie (2.59 ± 0.14) and Oriental Turtle Dove (3.10 ± 0.16) sounds (*p* < 0.001). Additionally, the reaction time to Oriental Magpie sounds was significantly shorter than that to Oriental Turtle Dove sounds (*z* = −4.34, *p* < 0.001; Figure 4). The reaction time increased with increasing group sizes of Azure‐winged Magpies (Figure 5).

**TABLE 2:** Responses of Azure‐winged magpies to different intruder sounds.

Variable	$x^{2}$	*df*	*p*
Reaction time			
Playback type	193.56	3	< 0.001
Group size	32.30	1	< 0.001
Response intensity			
Playback type	56.80	3	< 0.001
Group size	0.01	1	0.980

**FIGURE 6:**
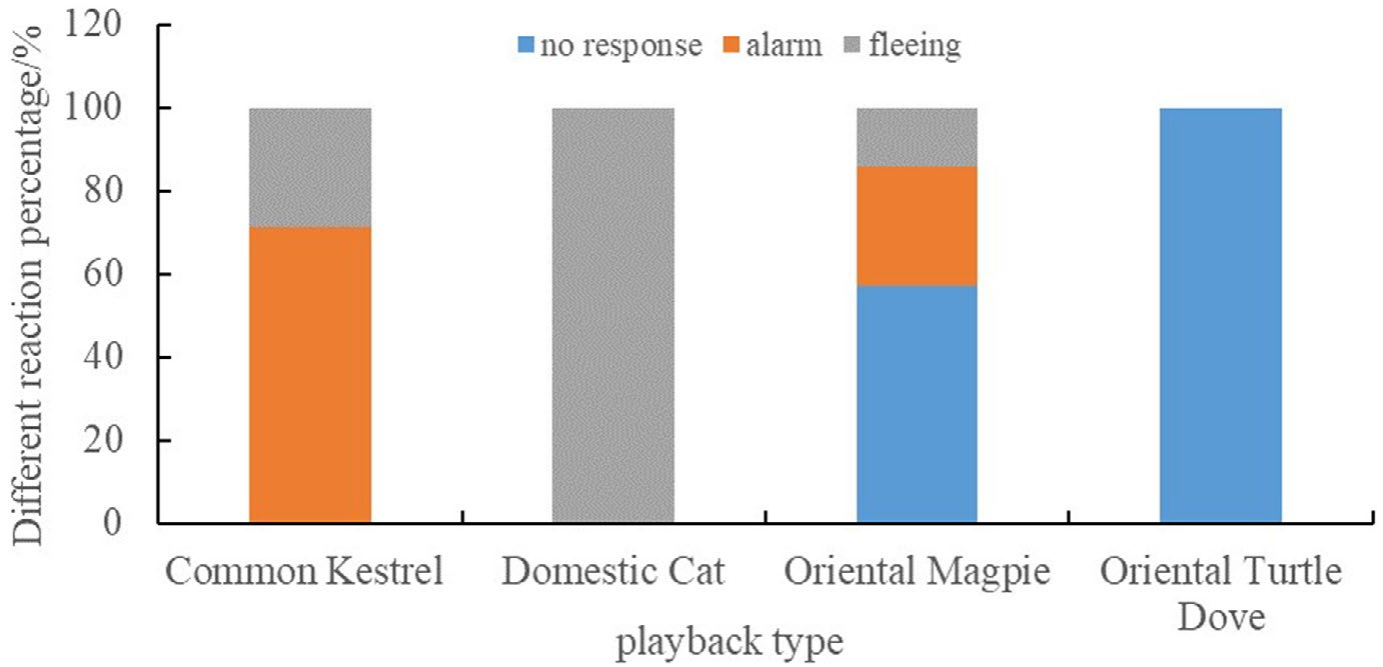
Responses of Azure‐winged magpies to different types of playback sounds.

The CLMM results indicated that the different types of playback sounds affected the response intensity of Azure‐winged Magpies significantly ($x^{2}$ = 56.80, *p* < 0.001; see Table 2). However, group size did not have a significant effect on the response intensity (*p* > 0.05; see Table 2). Post hoc pairwise comparisons showed that the response intensity of Azure‐winged Magpies to Common Kestrel sounds was significantly weaker than that to Domestic Cat sounds (*z* = −4.04, *p* < 0.001). Furthermore, the response intensities to Common Kestrel and Domestic Cat sounds were significantly greater than those to Oriental Magpie and Oriental Turtle Dove sounds (*p* < 0.001). Additionally, the response intensity to Oriental Magpie sounds was greater than that to Oriental Turtle Dove sounds (*z* = 2.71, *p* = 0.01; see Figure 6). In addition, we did not find any effect of the order effect on the response variable of the magpie (All *p* > 0.05).

## 4 Discussion

The results of this study indicate that during the non‐breeding season, Azure‐winged Magpies can recognize different types of predators through both visual and auditory cues. Both recognition cues accurately distinguished threat levels, prompting Azure‐winged Magpies to adjust their foraging behavior accordingly. Azure‐winged Magpies exhibited stronger responses to Domestic Cats than to other species.

Consistent with our predictions, in the specimen experiment, Azure‐winged Magpies exhibited a longer observation time and shorter foraging duration in response to the more frequently encountered Domestic Cat than to the Common Kestrel. This indicated that Azure‐winged Magpies perceived that Domestic Cats were a greater threat. This was consistent with the findings of Carlson, Pargeter, and Templeton (2017), showing that mixed flocks of Eurasian Blue Tits and Great Tits exhibited a stronger mobbing intensity towards the resident predator, Sparrowhawk, than towards the non‐resident predator Little Owl ( *Athene noctua* ), possibly due to direct encounter experience. The significantly longer observation time of Azure‐winged Magpies in response to the Oriental Magpie specimen than the Oriental Turtle Dove may be explained by the territorial and food competition between Oriental Magpies and Azure‐winged Magpies (Palomino, Carrascal, and Potti 2011). During observation, Azure‐winged Magpies may need more time to assess and weigh the competitive strength of both parties. Additionally, as the group size of Azure‐winged Magpies increased, both the observation time within 5 m and foraging time increased. Based on actual observations, we speculate that this may be because when facing uncertain risks, Azure‐winged Magpies increase their observation time or call in more individuals from the group to mitigate this uncertainty. Once safety is confirmed, more individuals join the foraging activity, extending the foraging time. This finding aligns with the many‐eyes hypothesis, which predicts that as group size increases, more individuals are available to scan for predators in the surrounding environment (Pulliam 1973). Therefore, individual foragers can spend less time being vigilant (and more time foraging) without compromising the ability of the group to detect threats (Lima 1995). This prediction has been confirmed in hundreds of studies (Hammer et al. 2023; McAlister and Hamilton 2022; Randler 2003).

The different response times of Azure‐winged Magpies to various playback sounds indicate varying levels of threat from different predators or non‐predators. As the apparent threat level increased, Azure‐winged Magpies exhibited stronger responses, similar to the results of the specimen experiment. This also aligns with the threat‐sensitive predator avoidance hypothesis. When the group size of Azure‐winged Magpies increases, the response time tends to increase, possibly because the risk of an individual being predated decreases as the group size increases (Duca, Brunelli, and Doherty Jr. 2019; Hammer et al. 2023; Bednekoff and Lima 1998). According to the “dilution effect hypothesis,” individuals in larger groups can tolerate predators coming closer before initiating flight (thereby spending more time on tasks like foraging) because the risk of predation is diluted with each additional conspecific nearby (Boland 2003; Cresswell 1994; Fernández‐Juricic and Schroeder 2003).

Additionally, even for the same stimulus type, there was considerable feeding behavior variation among individuals within the group. This is similar to the results of a study of domesticated Budgerigars ( *Melopsittacus undulatus* ) (Medina‐García, Jawor, and Wright 2017), which may be related to the personality of the magpie. Certain research studies indicate that personality influences the exploratory and anti‐predatory behavior of birds (Paulino, Nogueira‐Filho, and Nogueira 2018). As exploratory behavior increases, bolder individuals may face greater risks from predators, exhibit higher levels of aggression, and recover from stress relatively speedily. In contrast, shy individuals tend to be more cautious in the presence of predators (Carere et al. 2005; Carere and van Oers 2004). Additionally, anti‐predator behaviors in birds may be sex‐related; males generally show greater vigilance than females (Dávila et al. 2019). Furthermore, anti‐predator behavior may be linked to individual experience, those frequently exposed to predation pressure may exhibit relatively strong stress responses, but they may also habituate to human presence (Ellenberg, Mattern, and Seddon 2009; Ellenberg et al. 2007). Studies suggest that populations with varying personality types are less susceptible to environmental changes, displaying smaller density fluctuations near equilibrium, which enhances the carrying capacity (i.e., equilibrium population density) and productivity of the population (Bolnick et al. 2011; Sih 2004). Ultimately, the rapid co‐evolution of predators and prey is often mediated by behavior, and personality differences can significantly accelerate adaptive evolution (Sih, Bell, and Johnson 2004; Wolf and Weissing 2012). Further research is needed to examine the impact of personality on anti‐predator behaviors.

In the present study, it was difficult to establish consistent response variables between the playback experiment and the specimen experiment; therefore, no direct comparison was made between the two modes (visual and auditory signals). However, we suspect that predators' auditory cues may be more effective than their visual cues for magpies, for several reasons. First, there are many buildings and trees in the campus, which leads to the visual signal being hindered to some extent. On the contrary, the auditory signal is not affected, so that the auditory signal may have the advantage. Second, the predator specimens used in the present study were stationary, and magpies were able to see the predator, which allowed them to adjust their response to predator status. Conversely, when the experiment is replayed, the unseen threat poses a greater danger, because failure to respond immediately could put lives at risk. Third, according to our playback experiment, magpies react quickly and alarm or flee when playing back predator sounds, and when displaying a predator specimen, most of the magpies had a longer observation time. Therefore, auditory signals are potentially more effective for magpies on campus, but further verification is still needed. In addition, although we did not directly compare the two models, the same results were obtained, namely, the magpies were able to recognize different types of predators both visually and aurally, and the results were consistent.

Furthermore, because the experiment was conducted in a single, artificial habitat, the magpie may have adapted to human activities, reducing its sensitivity to frequent stressors such as humans, while remaining vigilant against actual predators such as domestic cats and kestrels (Cavalli et al. 2016). In experiments conducted in forested or rural areas, the magpies might adjust their behavior according to changing habitat conditions to prevent individual fitness loss (Sih, Ferrari, and Harris 2011). Specifically, in contrast with the university campus, the composition of predators is notably in a forest landscape where domestic cats are virtually absent and kestrels are relatively common, prompting a stronger response from the magpies (Bogrand, Neudorf, and Matich 2017; Duré Ruiz, Mariana, and Fernández 2017). In a rural landscape, the frequency of domestic cats would likely exceed that of kestrels, resulting in outcomes similar to those of this experiment. Future research should explore the flexibility of the magpies' anti‐predator behavior in more diverse environments.

In conclusion, the study results indicate that Azure‐winged Magpies can recognize different types of predators through both visual and auditory signals and adjust their foraging behavior according to the level of risk during the non‐breeding season. The findings contribute to a deeper understanding of the mechanisms underlying anti‐predator behavior in birds and provide new insights into predator–prey co‐evolution during the non‐breeding season, which can inform relevant conservation strategies and habitat management. However, whether anti‐predator behavior changes during the breeding season, how the magpies would respond to more predator types, and how they integrate multiple signals to identify predators and assess risks have not been comprehensively elucidated. These issues need to be studied further in future.

## Author Contributions

**Taijun Zuo:** data curation (equal), writing – original draft (lead). **Jiaojiao Wang:** conceptualization (lead), formal analysis (equal), funding acquisition (equal), writing – review and editing (equal). **Jiangnan Liu:** data curation (equal). **Jianping Liu:** funding acquisition (equal). **Qindong Zhou:** formal analysis (equal). **Jianhua Hou:** writing – review and editing (equal).

## Ethics Statement

The experiments reported here comply with the current laws of China. Experimental procedures were in agreement with the Animal Experiment Ethics Committee of Guizhou Normal University (No. 2022001).

## Conflicts of Interest

The authors declare no conflicts of interest.

## Supporting information


Data S1.

## Data Availability

The data related to this study has been submitted simultaneously as Supporting Information and is also available from the corresponding author upon reasonable request.
